# Supplementary material for: CVR-MRICloud: An online processing tool for CO2-inhalation and resting-state cerebrovascular reactivity (CVR) MRI data
Source: PLoS One. 2022 Sep 28;17(9):e0274220. doi: 10.1371/journal.pone.0274220 (PMC9518872; doi:10.1371/journal.pone.0274220)
Supplement: S1 Fig — The motion curve was in foot-head (FH) direction obtained using spm_realign.m function. (DOCX) [file pone.0274220.s001.docx]

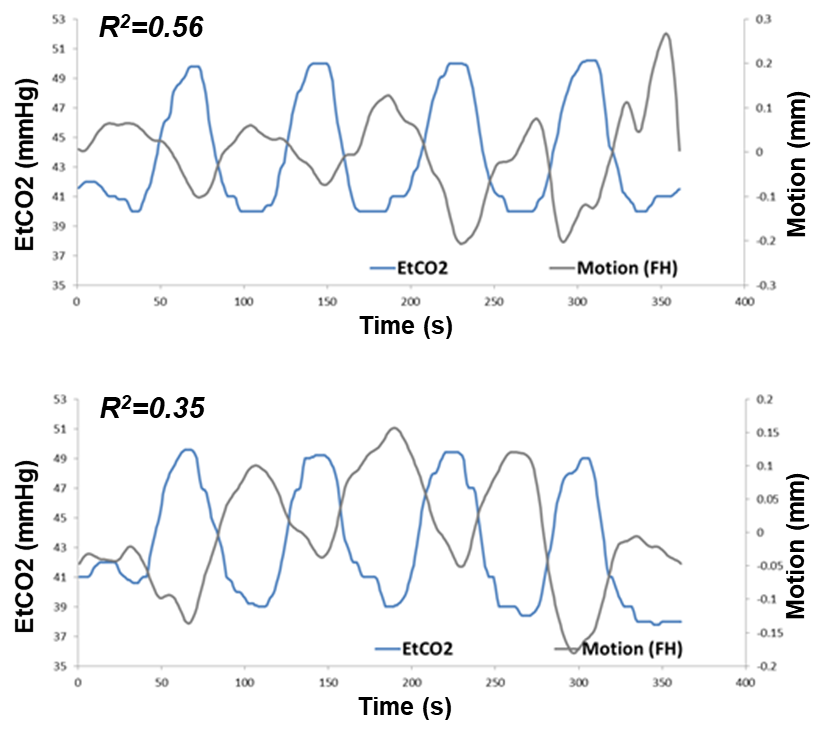


**Supplemental Figure S1:** EtCO2 curves and motion curves from two healthy subjects with high R^2^ values. The motion curve was in foot-head (FH) direction obtained using spm_realign.m function.
